# Log-convexity and the overpartition function

**DOI:** 10.1007/s11139-022-00578-0

**Published:** 2022-05-13

**Authors:** Gargi Mukherjee

**Affiliations:** Institute for Algebra, Johannes Kepler University, Altenberger Strasse 69, 4040 Linz, Austria

**Keywords:** Log-convexity, Overpartitions, Primary 05A20, 11N37

## Abstract

Let $\overline{p}(n)$ denote the overpartition function. In this paper, we obtain an inequality for the sequence $\Delta ^{2}\log \ \root n-1 \of {\overline{p}(n-1)/(n-1)^{\alpha }}$ which states that

$$\begin{aligned}&\log \biggl (1+\frac{3\pi }{4n^{5/2}}-\frac{11+5\alpha }{n^{11/4}}\biggr )< \Delta ^{2} \log \ \root n-1 \of {\overline{p}(n-1)/(n-1)^{\alpha }}\\&< \log \biggl (1+\frac{3\pi }{4n^{5/2}}\biggr ) \ \ \text {for}\ n \ge N(\alpha ), \end{aligned}$$

where $\alpha$ is a non-negative real number, $N(\alpha )$ is a positive integer depending on $\alpha$, and $\Delta$ is the difference operator with respect to *n*. This inequality consequently implies $\log$-convexity of $\bigl \{\root n \of {\overline{p}(n)/n}\bigr \}_{n \ge 19}$ and $\bigl \{\root n \of {\overline{p}(n)}\bigr \}_{n \ge 4}$. Moreover, it also establishes the asymptotic growth of $\Delta ^{2} \log \ \root n-1 \of {\overline{p}(n-1)/(n-1)^{\alpha }}$ by showing $\underset{n \rightarrow \infty }{\lim } \Delta ^{2} \log \ \root n \of {\overline{p}(n)/n^{\alpha }} = \dfrac{3 \pi }{4 n^{5/2}}.$

## Introduction

An overpartition of *n* is a non-increasing sequence of natural numbers whose sum is *n* in which the first occurrence of a number may be overlined and $\overline{p}(n)$ denotes the number of overpartitions of *n*. For convenience, define $\overline{p}(0)=1$. For example, there are 8 overpartitions of 3 enumerated by $3, \overline{3}, 2+1, \overline{2}+1, 2+\overline{1}, \overline{2}+\overline{1},1+1+1, \overline{1}+1+1$. Systematic study of overpartition began with the work of Corteel and Lovejoy [4], although it has been studied under different nomenclatures that date back to MacMahon. Analogous to Hardy–Ramanujan–Rademacher formula for partition function (cf. [7, 10]), Zuckerman [13] gave a formula for $\overline{p}(n)$ that reads

1.1 $$\begin{aligned} \overline{p}(n)=\frac{1}{2\pi }\underset{2 \not \mid k}{\sum _{k=1}^{\infty }}\sqrt{k}\underset{(h,k)=1}{\sum _{h=0}^{k-1}}\dfrac{\omega (h,k)^2}{\omega (2h,k)}e^{-\frac{2\pi i n h}{k}}\dfrac{d}{dn}\biggl (\dfrac{\sinh \frac{\pi \sqrt{n}}{k}}{\sqrt{n}}\biggr ), \end{aligned}$$

where

$$\begin{aligned} \omega (h,k)=\text {exp}\Biggl (\pi i \sum _{r=1}^{k-1}\dfrac{r}{k}\biggl (\dfrac{hr}{k}-\biggl \lfloor \dfrac{hr}{k}\biggr \rfloor -\dfrac{1}{2}\biggr )\Biggr ) \end{aligned}$$

for positive integers *h* and *k*. In somewhat a similar spirit as Lehmer [8] obtained an error bound for the partition function, Engel [6] provided an error term for $\overline{p}(n)$

1.2 $$\begin{aligned} \overline{p}(n)=\frac{1}{2\pi }\underset{2 \not \mid k}{\sum _{k=1}^{N}}\sqrt{k}\underset{(h,k)=1}{\sum _{h=0}^{k-1}}\dfrac{\omega (h,k)^2}{\omega (2h,k)}e^{-\frac{2\pi i n h}{k}}\dfrac{d}{dn}\biggl (\dfrac{\sinh \frac{\pi \sqrt{n}}{k}}{\sqrt{n}}\biggr )+R_{2}(n,N), \end{aligned}$$

where

1.3 $$\begin{aligned} \bigl |R_{2}(n,N)\bigr |< \dfrac{N^{5/2}}{\pi n^{3/2}} \sinh \biggl (\dfrac{\pi \sqrt{n}}{N}\biggr ). \end{aligned}$$

A positive sequence $\{a_n\}_{n \ge 0}$ is called $\log$-convex if for $n \ge 1$,

$$\begin{aligned} a^2_n-a_{n-1}a_{n+1}\le 0, \end{aligned}$$

and it is called $\log$-concave if for $n \ge 1$,

$$\begin{aligned} a^2_n-a_{n-1}a_{n+1}\ge 0. \end{aligned}$$

Engel [6] proved that $\{\overline{p}(n)\}_{n \ge 2}$ is $\log$-concave by using the asymptotic formula (1.2) with $N=2$ followed by (1.3). Prior to Engel’s work on overpartitions, $\log$-concavity of partition function *p*(*n*) and its associated inequalities has been studied in a broad spectrum, for example see [1, 2], and [5]. Following the same line of studies, Liu and Zhang [9] proved a list of inequalities for overpartition function.

Sun [11] initiated the study on $\log$-convexity problems associated with *p*(*n*), later settled by Chen and Zheng [3, Theorem 1.1-1.2]. In a more general setting, Chen and Zheng studied $\log$-convexity of $\{\root n \of {p(n)/n^{\alpha }}\}_{n \ge n(\alpha )}$ (cf. [3, Theorem 1.3]). Moreover, they discovered the asymptotic growth of the sequence $\Delta ^2 \log \root n \of {p(n)}$ (cf. [3, Theorem 1.4]).

The main objective of this paper is to prove all the theorems [3, Theorem 1.1-1.4] but in the context of overpartitions. Our goal is to obtain a much more general inequality, given in Theorem 1.1, which at once implies [3, Theorem 1.1-1.4] for $\overline{p}(n)$, presented in Corollary 1.2-1.5. More explicitly, in Theorem 1.1, we get a somewhat symmetric upper and lower bound of $\root n \of {\overline{p}(n)/n^{\alpha }}$, as shown in (1.4). We note that the lower bound presented in (1.4) depicts a finer inequality than merely stating $\Delta ^{2} \log \root n \of {\overline{p}(n)/n^{\alpha }} > 0$ which implies $\log$-convexity. In another direction, we note that (1.4) readily suggests that $\dfrac{3\pi }{4}$ is the best possible constant so as to understand the asymptotic growth of $\Delta ^{2} \log \root n \of {\overline{p}(n)/n^{\alpha }}$, given in Corollary 1.5.

For $$\alpha \in {\mathbb {R}}_{\ge 0}$$, define $$r_{\alpha }(n):=\root n \of {\overline{p}(n)/n^{\alpha }}$$.

### Theorem 1.1

Let $$\alpha \in {\mathbb {R}}_{\ge 0}$$ and

$$\begin{aligned} N(\alpha ):= {\left\{ \begin{array}{ll} \max \Biggl \{\Bigl [\dfrac{3490}{\alpha }\Bigr ]+2,\Bigl \lceil \Bigl (\dfrac{4(11+5\alpha )}{3\pi }\Bigr )^4\Bigr \rceil ,5505\Biggr \} &{}\quad \text {if }\alpha \in {\mathbb {R}}_{>0},\\ {4522} &{}\quad \text {if }\alpha = 0.\\ \end{array}\right. } \end{aligned}$$

Then for $$n \ge N(\alpha )$$,

1.4 $$\begin{aligned} \log \biggl (1+\frac{3\pi }{4n^{5/2}}-\frac{11+5\alpha }{n^{11/4}}\biggr )< \Delta ^{2} \log r_{\alpha }(n-1) < \log \biggl (1+\frac{3\pi }{4n^{5/2}}\biggr ). \end{aligned}$$

### Corollary 1.2

The sequence $\bigl \{\root n \of {\overline{p}(n)/n^{\alpha }}\bigr \}_{n \ge N(\alpha )}$ is $\log$-convex.

### Proof

From (1.4), it is immediate that

$$\begin{aligned} \dfrac{r_{\alpha }(n+1)r_{\alpha }(n-1)}{r^2_{\alpha }(n)} > 1+\frac{3\pi }{4n^{5/2}}-\frac{11+5\alpha }{n^{11/4}}\ \ \text {for all}\ \ n \ge N(\alpha ). \end{aligned}$$

We finish the proof by observing that

$$\begin{aligned} 1+\frac{3\pi }{4n^{5/2}}-\frac{11+5\alpha }{n^{11/4}} >1\ \ \text {for all}\ \ n \ge N(\alpha ). \end{aligned}$$

$$\square $$

### Corollary 1.3

The sequences $\bigl \{\root n \of {\overline{p}(n)/n}\bigr \}_{n \ge 19}$ and $\bigl \{\root n \of {\overline{p}(n)}\bigr \}_{n \ge 4}$ are $\log$-convex.

### Proof

In order to prove $\bigl \{\root n \of {\overline{p}(n)/n}\bigr \}_{n \ge 19}$ and $\bigl \{\root n \of {\overline{p}(n)}\bigr \}_{n \ge 4}$ are $\log$-convex, after corollary 1.2, it remains to check numerically for $19\le n \le 5504$ and $4 \le n \le 4521$, which is done in ‘Mathematica’ interface. $\square$

### Corollary 1.4

For all $n \ge 2$, we have

1.5 $$\begin{aligned} \dfrac{\root n \of {\overline{p}(n)}}{\root n+1 \of {\overline{p}(n+1)}}\biggl (1+\frac{3\pi }{4n^{5/2}}\biggr ) > \dfrac{\root n-1 \of {\overline{p}(n-1)}}{\root n \of {\overline{p}(n)}}. \end{aligned}$$

### Proof

It is an immediate implication of (1.4) as it is only left over to verify (1.5) for $2\le n \le 4522$, which we did numerically in ‘Mathematica.’ $\square$

### Corollary 1.5

1.6 $$\begin{aligned} \lim _{n \rightarrow \infty } n^{5/2} \Delta ^{2} \log r_{\alpha }(n) = \frac{3 \pi }{4}. \end{aligned}$$

### Proof

Multiplying both the sides of (1.4) by $n^{5/2}$ and taking limit as *n* tends to infinity, we get (1.6). $\square$

## Proof of theorem 1.1

In this section, we give a proof of Theorem 1.1. First, we state the Lemma 2.1 [3, Lemma 2.1] of Chen and Zheng which will be useful in the proofs of Lemmas 2.2-2.4. These lemmas further direct to get upper bound and lower bound of $\Delta ^{2} \log r_{\alpha }(n)$, respectively, in Lemma 2.5 and 2.6, finally results (1.4).

### Lemma 2.1

[3, Lemma 2.1] Suppose *f*(*x*) has a continuous second derivative for $x \in [n-1,n+1]$. Then there exists $c \in (n-1,n+1)$ such that

2.1 $$\begin{aligned} \Delta ^2 f(n-1)=f(n+1)+f(n-1)-2f(n)=f''(c). \end{aligned}$$

If *f*(*x*) has an increasing second derivative, then

2.2 $$\begin{aligned} f''(n-1)<\Delta ^2 f(n-1) < f''(n+1). \end{aligned}$$

Conversely, if *f*(*x*) has a decreasing second derivative, then

2.3 $$\begin{aligned} f''(n+1)< \Delta ^2 f(n-1) < f''(n-1). \end{aligned}$$

We start by laying out a brief outline of Engel’s primary set up [6] for proving $\log$-concavity of $\{\overline{p}(n)\}_{n \ge 2}$. Setting $N=3$ in (1.2), we express $\overline{p}(n)$ as

2.4 $$\begin{aligned} \overline{p}(n) = \overline{T}(n)+\overline{R}(n), \end{aligned}$$

where

2.5 $$\begin{aligned} \overline{T}(n)= & {} \dfrac{\overline{c}}{\overline{\mu }(n)^2}\biggl (1-\dfrac{1}{\overline{\mu }(n)}\biggr )e^{\overline{\mu }(n)},\end{aligned}$$

2.6 $$\begin{aligned} \overline{R}(n)= & {} \dfrac{1}{8n}\biggl (1+\dfrac{1}{\overline{\mu }(n)}\biggr )e^{-\overline{\mu }(n)}+R_{2}(n,3) \end{aligned}$$

with $\overline{c}=\dfrac{\pi ^2}{8}$ and $\overline{\mu }(n)=\pi \sqrt{n}$. In order to estimate the upper and lower bound of $\Delta ^{2} \log r_{\alpha }(n-1)$, it is necessary for us to express $\Delta ^{2} \log r_{\alpha }(n-1)$ in the following form

2.7 $$\begin{aligned} \Delta ^{2} \log r_{\alpha }(n-1)= & {} \Delta ^2 \dfrac{1}{n-1} \log \overline{p}(n-1) - \alpha \ \Delta ^2 \dfrac{1}{n-1} \log (n-1)\nonumber \\= & {} \Delta ^2 \dfrac{1}{n-1} \log \overline{T}(n-1) + \Delta ^2 \dfrac{1}{n-1}\nonumber \\&\log \biggl (1+ \dfrac{\overline{R}(n-1)}{\overline{T}(n-1)}\biggr )- \alpha \ \Delta ^2 \dfrac{1}{n-1} \log (n-1).\nonumber \\ \end{aligned}$$

Define

2.8 $$\begin{aligned} \overline{E}(n-1) = \log \biggl (1+ \dfrac{\overline{R}(n-1)}{\overline{T}(n-1)}\biggr ) \end{aligned}$$

and rewrite (2.7) as

2.9 $$\begin{aligned}&\Delta ^{2} \log r_{\alpha }(n-1) = \Delta ^2 \dfrac{1}{n-1} \log \overline{T}(n-1) + \Delta ^2 \dfrac{1}{n-1} \overline{E}(n-1)\nonumber \\&- \alpha \ \Delta ^2 \dfrac{1}{n-1} \log (n-1) \end{aligned}$$

Therefore, in order to estimate $\Delta ^{2} \log r_{\alpha }(n-1)$, it is sufficient to estimate each of the three factors, appearing on the right-hand side of (2.9).

### Lemma 2.2

Let

2.10 $$\begin{aligned} \overline{G}_{1}(n)= & {} \dfrac{3\pi }{4(n+1)^{5/2}}-\dfrac{5 \log \overline{\mu }(n-1)}{(n-1)^3},\end{aligned}$$

2.11 $$\begin{aligned} \overline{G}_{2}(n)= & {} \dfrac{3\pi }{4(n-1)^{5/2}}-\dfrac{3 \log \overline{\mu }(n+1)}{(n+1)^3}+\dfrac{4}{(n-1)^3}. \end{aligned}$$

Then for $n \ge 2$, we have

2.12 $$\begin{aligned} \overline{G}_{1}(n)< \Delta ^2 \dfrac{1}{n-1} \log \overline{T}(n-1) < \overline{G}_{2}(n). \end{aligned}$$

### Proof

Using the definition of $\overline{T}(n)$ (2.5), we write

2.13 $$\begin{aligned} \Delta ^2 \dfrac{1}{n-1} \log \overline{T}(n-1) = \sum _{i=1}^{4} \Delta ^2 \ \overline{g}_{i}(n-1), \end{aligned}$$

where

$$\begin{aligned} \overline{g}_1(n)= & {} \dfrac{\overline{\mu }(n)}{n},\\ \overline{g}_2(n)= & {} -\dfrac{3 \log \ \overline{\mu }(n)}{n},\\ \overline{g}_3(n)= & {} \dfrac{\log \ (\overline{\mu }(n)-1)}{n},\\ \text {and} \ \ \overline{g}_4(n)= & {} \dfrac{\log \overline{c}}{n}. \end{aligned}$$

It can be easily checked that for $n \ge 3$, $\overline{g}^{'''}_1(n)<0$, $\overline{g}^{'''}_2(n)>0$, $\overline{g}^{'''}_3(n)<0$, and $\overline{g}^{'''}_4(n)<0$. As a consequence, for $n \ge 3$, $\overline{g}^{''}_1(n), \overline{g}^{''}_3(n)$, and $\overline{g}^{''}_4(n)$ are decreasing, whereas $\overline{g}^{''}_2(n)$ is increasing. Applying Lemma 2.1, we get for $i \in \{1,3,4\}$,

2.14 $$\begin{aligned} \overline{g}^{''}_i(n+1)< \Delta ^2 \ \overline{g}_{i}(n-1) < \overline{g}^{''}_i(n-1) \end{aligned}$$

and

2.15 $$\begin{aligned} \overline{g}^{''}_2(n-1)< \Delta ^2 \ \overline{g}_{2}(n-1) < \overline{g}^{''}_2(n+1). \end{aligned}$$

From (2.13) and (2.14)-(2.15), we obtain for all $n \ge 3$,

2.16 $$\begin{aligned} \Delta ^2 \dfrac{1}{n-1} \log \overline{T}(n-1) < \overline{g}^{''}_1(n-1)+\overline{g}^{''}_2(n+1)+\overline{g}^{''}_3(n-1)+\overline{g}^{''}_4(n-1)\nonumber \\ \end{aligned}$$

and

2.17 $$\begin{aligned} \Delta ^2 \dfrac{1}{n-1} \log \overline{T}(n-1) > \overline{g}^{''}_1(n+1)+\overline{g}^{''}_2(n-1)+\overline{g}^{''}_3(n+1)+\overline{g}^{''}_4(n+1),\nonumber \\ \end{aligned}$$

where

2.18 $$\begin{aligned} \overline{g}^{''}_1(n)= & {} \dfrac{3\pi }{4n^{5/2}}, \end{aligned}$$

2.19 $$\begin{aligned} \overline{g}^{''}_2(n)= & {} \dfrac{9}{2n^3}-\dfrac{6\log \overline{\mu }(n)}{n^3}, \end{aligned}$$

2.20 $$\begin{aligned} \overline{g}^{''}_3(n)= & {} \dfrac{2 \log (\overline{\mu }(n)-1)}{n^3}-\dfrac{5\pi }{4n^{5/2}(\overline{\mu }(n)-1)}-\dfrac{\pi ^2}{4n^2 (\overline{\mu }(n)-1)^2}, \end{aligned}$$

2.21 $$\begin{aligned} \text {and}\ \ \overline{g}^{''}_4(n)= & {} \dfrac{2 \log \overline{c}}{n^3}. \end{aligned}$$

We first estimate the upper bound of $\Delta ^2 \dfrac{1}{n-1} \log \overline{T}(n-1)$ by (2.16) and (2.18)-(2.21).

2.22 $$\begin{aligned} \Delta ^2 \dfrac{1}{n-1} \log \overline{T}(n-1)< & {} \dfrac{3\pi }{4(n-1)^{5/2}}+\dfrac{9}{2(n+1)^3}\nonumber \\&-\dfrac{6\log \overline{\mu }(n+1)}{(n+1)^3}\nonumber \\&+\dfrac{2 \log (\overline{\mu }(n-1)-1)}{(n-1)^3}-\dfrac{5\pi }{4(n-1)^{5/2}(\overline{\mu }(n-1)-1)}\nonumber \\&-\dfrac{\pi ^2}{4(n-1)^2 (\overline{\mu }(n-1)-1)^2}\nonumber \\&+\dfrac{2 \log \overline{c}}{(n-1)^3}\nonumber \\&=\dfrac{3\pi }{4(n-1)^{5/2}}+\overline{U}_{1}(n)+\overline{U}_{2}(n), \end{aligned}$$

where

2.23 $$\begin{aligned} \overline{U}_{1}(n)= & {} -\dfrac{6\log \overline{\mu }(n+1)}{(n+1)^3}+\dfrac{2 \log (\overline{\mu }(n-1)-1)}{(n-1)^3} \end{aligned}$$

2.24 $$\begin{aligned} \text {and}\ \ \overline{U}_{2}(n)= & {} \dfrac{9}{2(n+1)^3}\nonumber \\&-\dfrac{5\pi }{4(n-1)^{5/2}(\overline{\mu }(n-1)-1)}-\dfrac{\pi ^2}{4(n-1)^2 (\overline{\mu }(n-1)-1)^2}+\dfrac{2 \log \overline{c}}{(n-1)^3}.\nonumber \\ \end{aligned}$$

It can be easily checked that for all $n \ge 2$,

2.25 $$\begin{aligned} \overline{U}_{2}(n) < \dfrac{4}{(n-1)^3}. \end{aligned}$$

For an upper bound of $\overline{U}_{1}(n)$, we observe that for all $n \ge 15$,

2.26 $$\begin{aligned} \dfrac{2}{(n-1)^3}< \dfrac{3}{(n+1)^3} \ \ \text {and}\ \ \log (\overline{\mu }(n)-1) < \log \overline{\mu }(n+1), \end{aligned}$$

that is,

2.27 $$\begin{aligned} \dfrac{2 \log (\overline{\mu }(n-1)-1)}{(n-1)^3} < \dfrac{3 \log \overline{\mu }(n+1)}{(n+1)^3}. \end{aligned}$$

Consequently for $n \ge 15$ we get,

2.28 $$\begin{aligned} \overline{U}_{1}(n) < -\dfrac{3\log \overline{\mu }(n+1)}{(n+1)^3}. \end{aligned}$$

Invoking (2.25) and (2.28) into (2.22), we have for $n \ge 15$,

2.29 $$\begin{aligned} \Delta ^2 \dfrac{1}{n-1} \log \overline{T}(n-1) < \dfrac{3\pi }{4(n-1)^{5/2}}-\dfrac{3\log \overline{\mu }(n+1)}{(n+1)^3}+\dfrac{4}{(n-1)^3}=\overline{G}_2(n). \end{aligned}$$

For lower bound of $\Delta ^2 \dfrac{1}{n-1} \log \overline{T}(n-1)$, using (2.17) and (2.18)-(2.21) we obtain

2.30 $$\begin{aligned} \begin{aligned} \Delta ^2 \dfrac{1}{n-1} \log \overline{T}(n-1) >&\dfrac{3\pi }{4(n+1)^{5/2}}+\dfrac{9}{2(n-1)^3}-\dfrac{6\log \overline{\mu }(n-1)}{(n-1)^3}\\&+\dfrac{2 \log (\overline{\mu }(n+1)-1)}{(n+1)^3}-\dfrac{5\pi }{4(n+1)^{5/2}(\overline{\mu }(n+1)-1)}\\&-\dfrac{\pi ^2}{4(n+1)^2 (\overline{\mu }(n+1)-1)^2}\\&+\dfrac{2 \log \overline{c}}{(n+1)^3}\\&=\dfrac{3\pi }{4(n+1)^{5/2}}+\overline{L}_{1}(n)+\overline{L}_{2}(n), \end{aligned} \end{aligned}$$

where

2.31 $$\begin{aligned} \overline{L}_{1}(n)= & {} -\dfrac{6\log \overline{\mu }(n-1)}{(n-1)^3}+\dfrac{2 \log (\overline{\mu }(n+1)-1)}{(n+1)^3} \end{aligned}$$

2.32 $$\begin{aligned} \text {and}\ \ \overline{L}_{2}(n)= & {} \dfrac{9}{2(n-1)^3}\nonumber \\&-\dfrac{5\pi }{4(n+1)^{5/2}(\overline{\mu }(n+1)-1)}-\dfrac{\pi ^2}{4(n+1)^2 (\overline{\mu }(n+1)-1)^2}+\dfrac{2 \log \overline{c}}{(n+1)^3}. \end{aligned}$$

Similarly as before, one can check that for $n \ge 9$,

2.33 $$\begin{aligned} \overline{L}_{2}(n)>0 \ \ \ \text {and}\ \ \ \overline{L}_{1}(n) > -\dfrac{5 \log \overline{\mu }(n-1)}{(n-1)^3}. \end{aligned}$$

(2.30) and (2.33) yield for $n \ge 9$,

2.34 $$\begin{aligned} \Delta ^2 \dfrac{1}{n-1} \log \overline{T}(n-1) > \dfrac{3\pi }{4(n+1)^{5/2}}-\dfrac{5 \log \overline{\mu }(n-1)}{(n-1)^3}= \overline{G}_{1}(n). \end{aligned}$$

(2.29) and (2.34) together imply (2.12) for $n \ge 15$. We finish the proof by checking (2.12) numerically for $2\le n \le 14$. $\square$

### Lemma 2.3

For $$n \ge 38$$,

2.35 $$\begin{aligned} \bigl |\Delta ^2 \ \dfrac{1}{n-1} \overline{E}(n-1) \bigr |<\dfrac{5}{n-1}e^{-\dfrac{\overline{\mu }(n-1)}{12}}. \end{aligned}$$

### Proof

Using (2.8), we get for $n \ge 2$,

2.36 $$\begin{aligned}&\Delta ^2 \ \dfrac{1}{n-1} \overline{E}(n-1) = \dfrac{1}{n+1} \log (1+\overline{e}(n+1)) \nonumber \\&-\dfrac{2}{n} \log (1+\overline{e}(n))+\dfrac{1}{n-1} \log (1+\overline{e}(n-1)), \end{aligned}$$

where

$$\begin{aligned} \overline{e}(n) = \dfrac{\overline{R}(n)}{\overline{T}(n)}. \end{aligned}$$

Taking absolute value of $\Delta ^2 \ \dfrac{1}{n-1} \overline{E}(n-1)$ in (2.36), we obtain for all $n \ge 2$,

2.37 $$\begin{aligned}&\bigl |\Delta ^2 \ \dfrac{1}{n-1} \overline{E}(n-1) \bigr | \le \dfrac{1}{n+1} |\log (1+\overline{e}(n+1))|\nonumber \\&+\dfrac{2}{n} |\log (1+\overline{e}(n))|+\dfrac{1}{n-1} |\log (1+\overline{e}(n-1))|. \end{aligned}$$

Therefore, it is enough to estimate $|\overline{e}(n)|$. Before proceeding to estimate, let us recall the bound of Engel [6](cf. (1.3)) for $N=3$ that yields for $n \ge 1$,

2.38 $$\begin{aligned} |R_{2}(n,3)|< \dfrac{9 \sqrt{3}}{2 n \ \overline{\mu }(n)} e^{\overline{\mu }(n)/3} \end{aligned}$$

by making use of the fact that $\sinh (x) < \dfrac{e^x}{2}$ for $x>0$. Recalling the definitions in (2.5)-(2.6), we obtain

2.39 $$\begin{aligned} |\overline{e}(n)|= & {} \Biggl |\dfrac{\biggl (1+\dfrac{1}{\overline{\mu }(n)}\biggr )}{\biggl (1-\dfrac{1}{\overline{\mu }(n)}\biggr )}e^{- 2\overline{\mu }(n)}+ \dfrac{R_{2}(n,3)}{\dfrac{1}{8n}\biggl (1-\dfrac{1}{\overline{\mu }(n)}\biggr )e^{\overline{\mu }(n)}}\Biggr |\nonumber \\\le & {} \dfrac{\biggl (1+\dfrac{1}{\overline{\mu }(n)}\biggr )}{\biggl (1-\dfrac{1}{\overline{\mu }(n)}\biggr )}e^{- 2\overline{\mu }(n)} + \dfrac{36\sqrt{3}}{\overline{\mu }(n) \biggl (1-\dfrac{1}{\overline{\mu }(n)}\biggr )}e^{- 2\overline{\mu }(n)/3}\ \ \ (\text {by}\ (2.38))\nonumber \\= & {} \dfrac{e^{- 2\overline{\mu }(n)/3}}{\overline{\mu }(n)-1}\Biggl [\bigl (\overline{\mu }(n)+1\bigr )e^{- 4\overline{\mu }(n)/3}+36\sqrt{3}\Biggr ]\nonumber \\= & {} \dfrac{e^{- \overline{\mu }(n)/12}}{\overline{\mu }(n)-1}\Biggl [\Bigl (\bigl (\overline{\mu }(n)+1\bigr )e^{- 4\overline{\mu }(n)/3}+36\sqrt{3}\Bigr )e^{- \overline{\mu }(n)/2}\Biggr ]e^{- \overline{\mu }(n)/12}. \end{aligned}$$

It can be easily checked that

2.40 $$\begin{aligned} \dfrac{e^{- \overline{\mu }(n)/12}}{\overline{\mu }(n)-1} < 1 \ \ \ \text {for all}\ n \ge 1 \end{aligned}$$

and

2.41 $$\begin{aligned} \Bigl (\bigl (\overline{\mu }(n)+1\bigr )e^{- 4\overline{\mu }(n)/3}+36\sqrt{3}\Bigr )e^{- \overline{\mu }(n)/2} < 1 \ \ \ \text {for all}\ n \ge 7. \end{aligned}$$

Invoking (2.40) and (2.41) into (2.39), we obtain for $n \ge 7$

2.42 $$\begin{aligned} |\overline{e}(n)| < e^{- \overline{\mu }(n)/12} \end{aligned}$$

and consequently for $n \ge 38$,

2.43 $$\begin{aligned} e^{- \overline{\mu }(n)/12} < \dfrac{1}{5}. \end{aligned}$$

Putting together (2.42) and (2.43), we get for all $n \ge 38$,

2.44 $$\begin{aligned} |\overline{e}(n)|<\dfrac{1}{5}. \end{aligned}$$

Next we note that for all $n \ge 38$,

2.45 $$\begin{aligned} |\log (1+ \overline{e}(n))| \le \dfrac{|\overline{e}(n)|}{1-|\overline{e}(n)|} < \dfrac{5}{4} \ |\overline{e}(n)| \end{aligned}$$

because of the fact that, for $|x|<1$,

$$\begin{aligned} |\log (1+x)|< \dfrac{|x|}{1-|x|}. \end{aligned}$$

From (2.37) and (2.45), we obtain for $n \ge 38$,

2.46 $$\begin{aligned} \bigl |\Delta ^2 \ \dfrac{1}{n-1} \overline{E}(n-1) \bigr | < \dfrac{5}{4} \Bigl (\dfrac{|\overline{e}(n+1)|}{n+1}+2 \dfrac{|\overline{e}(n)|}{n}+\dfrac{|\overline{e}(n-1)|}{n-1}\Bigr ). \end{aligned}$$

Plugging (2.42) into (2.46), we have for $n \ge 38$,

2.47 $$\begin{aligned} \bigl |\Delta ^2 \ \dfrac{1}{n-1} \overline{E}(n-1) \bigr |< & {} \dfrac{5}{4} \Bigl (\dfrac{e^{-\overline{\mu }(n+1)/12}}{n+1}+2 \dfrac{e^{-\overline{\mu }(n)/12}}{n}+\dfrac{e^{-\overline{\mu }(n-1)/12}}{n-1}\Bigr )\nonumber \\< & {} \dfrac{5}{n-1} e^{-\overline{\mu }(n-1)/12} \end{aligned}$$

because the sequence $\Bigl \{\dfrac{1}{n}\ e^{-\overline{\mu }(n)/12}\Bigr \}_{n \ge 1}$ is decreasing. $\square$

### Lemma 2.4

For $$\alpha \in {\mathbb {R}}_{>0}$$ and $$n \ge 7$$,

2.48 $$\begin{aligned}&-\dfrac{2\alpha \log (n-1)}{(n-1)^3}+\dfrac{3\alpha }{(n-1)^3}< -\alpha \ \Delta ^2 \dfrac{1}{n-1} \log (n-1)<-\dfrac{2\alpha \log (n+1)}{(n+1)^3}\nonumber \\&+\dfrac{3\alpha }{(n+1)^3}. \end{aligned}$$

### Proof

We observe that, for $n \ge 7$,

$$\begin{aligned} \Biggl (-\dfrac{\log n}{n}\Biggr )^{'''}= -\dfrac{11}{n^4}+\dfrac{6 \log n}{n^4}>0. \end{aligned}$$

Setting $f(n):= -\dfrac{\log n}{n}$ and applying Lemma 2.1, we obtain for $n \ge 7$,

2.49 $$\begin{aligned}&-\dfrac{2\log (n-1)}{(n-1)^3}+\dfrac{3}{(n-1)^3}< -\Delta ^2 \dfrac{1}{n-1} \log (n-1) < -\dfrac{2\log (n+1)}{(n+1)^3}\nonumber \\&+\dfrac{3}{(n+1)^3}. \end{aligned}$$

Since $\alpha$ is a positive real number, from (2.49), we obtain (2.48). $\square$

### Lemma 2.5

For $$\alpha \in {\mathbb {R}}_{\ge 0}$$ and $$n \ge 4021$$,

2.50 $$\begin{aligned} \Delta ^{2} \log r_{\alpha }(n-1) < \log \biggl (1+\frac{3\pi }{4n^{5/2}}\biggr ). \end{aligned}$$

### Proof

Using (2.12), (2.35), and (2.48) into (2.9), we obtain for $n \ge 38$,

2.51 $$\begin{aligned} \Delta ^{2} \log r_{\alpha }(n-1) < \overline{G}_2(n)+\dfrac{5}{n-1}e^{-\dfrac{\overline{\mu }(n-1)}{12}}-\dfrac{2\alpha \log (n+1)}{(n+1)^3}+\dfrac{3\alpha }{(n+1)^3}.\nonumber \\ \end{aligned}$$

Note that for all $n \ge 4$,

2.52 $$\begin{aligned} -\dfrac{2\alpha \log (n+1)}{(n+1)^3}+\dfrac{3\alpha }{(n+1)^3} \le 0 \end{aligned}$$

and for $$n \ge 4021$$,

2.53 $$\begin{aligned} \dfrac{5}{n-1}e^{-\dfrac{\overline{\mu }(n-1)}{12}} < \dfrac{5}{(n-1)^3}. \end{aligned}$$

Therefore from (2.52)-(2.53), for all $n \ge 4021$, it follows that

2.54 $$\begin{aligned} \Delta ^{2} \log r_{\alpha }(n-1) < \dfrac{3\pi }{4(n-1)^{5/2}}-\dfrac{3 \log \overline{\mu }(n+1)}{(n+1)^3}+\dfrac{9}{(n-1)^3}. \end{aligned}$$

Apparently, for all $n \ge 93$,

2.55 $$\begin{aligned} \dfrac{3\pi }{4(n-1)^{5/2}}-\dfrac{3 \log \overline{\mu }(n+1)}{(n+1)^3}+\dfrac{9}{(n-1)^3} < \dfrac{3\pi }{4n^{5/2}}-\dfrac{9\pi ^2}{32n^{5}}. \end{aligned}$$

Using the fact that for $x >0$, $\log (1+x) > x-\dfrac{x^2}{2}$, from (2.54) and (2.55), we finally arrive at

2.56 $$\begin{aligned} \Delta ^{2} \log r_{\alpha }(n-1) < \log \Bigl (1+\dfrac{3\pi }{4n^{5/2}}\Bigr ). \end{aligned}$$

$$\square $$

### Lemma 2.6

For $$\alpha >0$$ and $$n \ge \max \Biggl \{\Bigl [\dfrac{3490}{\alpha }\Bigr ]+2,\Bigl \lceil \Bigl (\dfrac{4(11+5\alpha )}{3\pi }\Bigr )^4\Bigr \rceil ,5505\Biggr \}$$,

2.57 $$\begin{aligned} \Delta ^{2} \log r_{\alpha }(n-1) > \log \biggl (1+\frac{3\pi }{4n^{5/2}}-\frac{11+5\alpha }{n^{11/4}}\biggr ). \end{aligned}$$

### Proof

Using (2.12), (2.35), and (2.48) into (2.9), we obtain for $n \ge 38$,

2.58 $$\begin{aligned} \Delta ^{2} \log r_{\alpha }(n-1) > \overline{G}_1(n)-\dfrac{5}{n-1}e^{-\dfrac{\overline{\mu }(n-1)}{12}}-\dfrac{2\alpha \log (n-1)}{(n-1)^3}+\dfrac{3\alpha }{(n-1)^3}.\nonumber \\ \end{aligned}$$

It is easy to check that for $n \ge \text {max} \Bigl \{\Bigl [\dfrac{3490}{\alpha }\Bigr ]+2,4522\Bigr \}:=N_1(\alpha )$,

2.59 $$\begin{aligned} -\dfrac{5}{n-1}e^{-\dfrac{\overline{\mu }(n-1)}{12}}+\dfrac{3\alpha }{(n-1)^3}> \dfrac{3\alpha }{(n-1)^3}-\dfrac{10470}{(n-1)^4}>0. \end{aligned}$$

Therefore for all $n \ge N_1(\alpha )$,

2.60 $$\begin{aligned} \Delta ^{2} \log r_{\alpha }(n-1) > \dfrac{3\pi }{4(n+1)^{5/2}}-\dfrac{5 \log \overline{\mu }(n-1)}{(n-1)^3}-\dfrac{2\alpha \log (n-1)}{(n-1)^3}. \end{aligned}$$

It is immediate that for $n \ge 11$,

2.61 $$\begin{aligned} \log \overline{\mu }(n-1)< \log (n-1) \end{aligned}$$

and for $$n \ge 5505$$,

2.62 $$\begin{aligned} \log (n-1) < (n-1)^{1/4}. \end{aligned}$$

Putting (2.61) and (2.62) into (2.60), we obtain for $n \ge \text {max}\{N_1(\alpha ),5505\}$,

2.63 $$\begin{aligned} \Delta ^{2} \log r_{\alpha }(n-1)> & {} \dfrac{3\pi }{4(n+1)^{5/2}} -\dfrac{5+2\alpha }{(n-1)^{11/4}}. \end{aligned}$$

It remains to show that

2.64 $$\begin{aligned} \dfrac{3\pi }{4(n+1)^{5/2}} -\dfrac{5+2\alpha }{(n-1)^{11/4}} > \dfrac{3\pi }{4n^{5/2}} -\dfrac{11+5\alpha }{n^{11/4}}. \end{aligned}$$

For $n \ge \ \text {max}\Biggl \{\Biggl \lceil \Biggr (\dfrac{15 \pi }{8 (\alpha +1)}\Biggr )^{4/3}\Biggr \rceil ,5\Biggr \} := N_2(\alpha )$, it follows that

2.65 $$\begin{aligned} \dfrac{11+5\alpha }{n^{11/4}}-\dfrac{5+2\alpha }{(n-1)^{11/4}}\underset{n \ge 5}{>} \dfrac{1+\alpha }{n^{11/4}}>\dfrac{15\pi }{8 n^{7/2}} \underset{n \ge 1}{>} \dfrac{3\pi }{4}\Biggl (\dfrac{1}{n^{5/2}}-\dfrac{1}{(n+1)^{5/2}}\Biggr ). \end{aligned}$$

From (2.63) and (2.64), we obtain for $n \ge \ \text {max}\Bigl \{N_1(\alpha ), N_2(\alpha ), 5505\Bigr \}$,

2.66 $$\begin{aligned} \Delta ^{2} \log r_{\alpha }(n-1) > \dfrac{3\pi }{4n^{5/2}} -\dfrac{11+5\alpha }{n^{11/4}}. \end{aligned}$$

It is easy to check that for $n \ge \Biggl \lceil \Bigl (\dfrac{4(11+5\alpha )}{3\pi }\Bigr )^4\Biggr \rceil :=N_3(\alpha )$,

2.67 $$\begin{aligned} \dfrac{3\pi }{4n^{5/2}} -\dfrac{11+5\alpha }{n^{11/4}}>0 \end{aligned}$$

and using the fact that for $x>0$, $x>\log (1+x)$, we finally get for $n \ge \text {max}\{N_1(\alpha ),N_3(\alpha ),5505\}$ $\Bigl (\text {since}, N_3(\alpha )> N_2(\alpha ) \ \text {for}\ \alpha >0\Bigr )$,

2.68 $$\begin{aligned} \Delta ^{2} \log r_{\alpha }(n-1) > \log \Bigl (1+\dfrac{3\pi }{4n^{5/2}} -\dfrac{11+5\alpha }{n^{11/4}}\Bigr ). \end{aligned}$$

$$\square $$

### Proof of Theorem 1.1

For $\alpha \in {\mathbb {R}}_{>0}$, from (2.50) and (2.57) we obtain for all $n \ge \ \text {max} \Biggl \{\Bigl [\dfrac{3490}{\alpha }\Bigr ]+2,\Bigl \lceil \Bigl (\dfrac{4(11+5\alpha )}{3\pi }\Bigr )^4\Bigr \rceil ,5505\Biggr \}$,

2.69 $$\begin{aligned} \log \biggl (1+\frac{3\pi }{4n^{5/2}}-\frac{11+5\alpha }{n^{11/4}}\biggr )<\Delta ^{2} \log r_{\alpha }(n-1) < \log \biggl (1+\frac{3\pi }{4n^{5/2}}\biggr ). \end{aligned}$$

For $\alpha =0$, we have already seen that for $n \ge 4021$,

2.70 $$\begin{aligned} \Delta ^{2} \log r_{\alpha }(n-1) < \log \biggl (1+\frac{3\pi }{4n^{5/2}}\biggr ). \end{aligned}$$

For $\alpha =0$, using (2.12) and (2.35) into (2.9), we get for $n \ge 38$,

2.71 $$\begin{aligned} \Delta ^{2} \log r_{\alpha }(n-1) > \overline{G}_1(n)-\dfrac{5}{n-1}e^{-\dfrac{\overline{\mu }(n-1)}{12}}. \end{aligned}$$

Following the same approach, it can be checked that for $n \ge 4522$,

2.72 $$\begin{aligned} -\dfrac{5}{n-1}e^{-\dfrac{\overline{\mu }(n-1)}{12}} > -\dfrac{10470}{(n-1)^4} \end{aligned}$$

and consequently for $n \ge 476$,

2.73 $$\begin{aligned} \overline{G}_1(n)-\dfrac{10470}{(n-1)^4}> \dfrac{3\pi }{4n^{5/2}}-\dfrac{11}{n^{11/4}}>0. \end{aligned}$$

So, for $\alpha =0$, by (2.71)-(2.73), we obtain for $n \ge 4522$,

2.74 $$\begin{aligned} \Delta ^{2} \log r_{\alpha }(n-1) > \log \biggl (1+\frac{3\pi }{4n^{5/2}}-\frac{11}{n^{11/4}}\biggr ). \end{aligned}$$

Putting (2.70) and (2.74), for $n \ge 4522$, it follows that

2.75 $$\begin{aligned} \log \biggl (1+\frac{3\pi }{4n^{5/2}}-\frac{11}{n^{11/4}}\biggr )<\Delta ^{2} \dfrac{1}{n-1} \log \overline{p}(n-1)< \log \biggl (1+\frac{3\pi }{4n^{5/2}}\biggr ).\nonumber \\ \end{aligned}$$

This finishes the proof. $\square$

## Conclusion

We conclude this paper by considering the following problem:

### Problem 3.1

Let $\alpha$ be a non-negative real number. Then for each $r\ge 1$, does there exists a positive integer $N(r,\alpha )$ so that for all $n \ge N(r,\alpha )$, one can obtain both upper bound and lower bound of $(-1)^r \Delta ^r \log r_{\alpha }(n)$ that finally shows the asymptotic growth of $(-1)^r \Delta ^r \log r_{\alpha }(n)$ as *n* tends to infinity?

For $r=2$, we have already seen that one can estimate $(-1)^r \Delta ^r \log r_{\alpha }(n)$, as given in Theorem 1.4 and its asymptotic growth is reflected in Corollary 1.5.

## Data Availability

We hereby confirm that Data sharing not applicable to this article as no datasets were generated or analyzed during the current study.
